# Times are changing: digitalisation in child and adolescent psychotherapy

**DOI:** 10.1007/s00787-020-01610-8

**Published:** 2020-07-31

**Authors:** Katharina Allgaier, Johanna Schmid, Karsten Hollmann, Pauline A. Reusch, Annette Conzelmann, Tobias J. Renner

**Affiliations:** 1 Department of Child and Adolescent Psychiatry, Psychosomatics and Psychotherapy, University Hospital of Psychiatry and Psychotherapy Tübingen, Center of Mental Health Tübingen, Osianderstraße 14, 72074 Tübingen, Germany; 2 Department of Psychology (Clinical Psychology II), PFH – Private University of Applied Sciences, Göttingen, Germany

Triggered by the SARS-CoV2-19 pandemic, the establishment of digital health interventions (DHI) to treat children and adolescents with psychiatric disorders has been pushed forward with an astonishing velocity. General contact restrictions forced psychotherapists to treat their patients via DHI, especially video conferences. During the first months of the pandemic, acceptance of DHI greatly improved and the extension of technical- and IT infrastructure was propelled.

DHI offer a broad spectrum of methods ranging from the augmentation of face-to-face therapy to stand-alone DHI. However, the accelerated DHI usage in psychotherapy prompts specific questions: For which groups of patients do evidence-based approaches exist? What are prerequisites and precautions? And: what are general advantages and pitfalls of DHI? This guest-editorial aims to outline the scientific state of research on DHI and to discuss practical aspects of DHI-related interventions in psychotherapy.

## Evidence-based DHI

Hollis and colleagues [1] summarized 21 former meta-analyses and reviews from the beginning of the development of DHI in the 1990s until the end of 2015 in their meta-review and additionally updated a systematic review analysing 30 randomized controlled trials (RCT) using DHI. Their review represents an outstanding comprehensive approach and has recently been complemented by a systematic literature search of our own work group [2], strictly following the criteria established in the Preferred Reporting Items for Systematic Reviews and Meta-Analyses (PRISMA) statement.

The work by Hollis and colleagues and our own search revealed that most robust evidence exists for interventions for anxiety disorders and mild to moderate depression (especially computerized cognitive behavioural therapy (cCBT) approaches).

RCT for ADHD in children and adolescents revealed rather heterogeneous results which might be explained by their highly heterogeneous approaches. The studies ranged from neurofeedback trainings to executive functioning trainings and interventions targeting everyday organization and planning skills. They show comparable (inconsistent) effects to nonpharmacological face-to-face therapy [1, 2].

Increasing evidence emerges for DHI targeting autism spectrum disorders which include DHI for children from preschool age to adolescence and their parents [1, 2]. DHI for children and adolescents with ASD involve parents more frequently than DHI for other diagnoses.

Studies investigating DHI for other diagnoses such as eating disorders, psychosis, obsessive compulsive disorders, dyslexia, coordination disorders, conduct disorders with disruptive behaviour, tic disorders, insomnia or trichotillomania in children and adolescents are rather scarce. But the few available studies show promising results, though more high-quality studies are needed for a profound evaluation of DHI for those disorders.

Looking at the technical aspects, most studies use personal computers, which offer the broadest range of applications, ranging from psychoeducation in serious games or in video or written modules over training of cognitive functions to video sessions [1, 2]. Smartphone usage is on the rise, allowing ambulatory assessment of mood or experience in everyday life [1, 2]. Tablets are mainly used for trainings to improve attention or social and communicative skills via apps [1, 2].

Most published DHI studies were conducted in the Western Hemisphere and Asia, with only few studies from South American and African countries [2]. Due to the advantages of DHI in providing treatment over extensive distances and an increasingly evolving IT-infrastructure for example in African countries, more use of DHI can be expected worldwide.

### Practical aspects in using DHI

Implementing DHI in clinical practice requires careful consideration of a variety of prerequisites and precautions [3]. Table 1 gives an overview of practical prerequisites, ranging from personal aspects over data security and regional legal frameworks to the relevance of instable therapeutic situations including suicidality. DHI provide unique benefits, as proven during the SARS-CoV2-19 lock-down, but also bring potential pitfalls. Table 2 gives an overview on aspects regarding accessibility, the implementation of interventions itself, the evidence regarding interventions as well as economic aspects [4].

**Table 1:** Prerequisites and precautions necessary for DHI

Field	Aspects to consider	
Competences of patients and therapists	Personal Preferences	For specific devices/programs
	Personal Skills	Competence to handle electronic device/software
	Confidence	In treatment effectiveness
Data security	Software requirements	Use of software approved for psychotherapy by respective health care authorities
		Software protects data from external access
	Data storage	Secure storage of patient data (including access control, encryption, and external backups) in accordance with requirements of the respective health care regulations
Implementation	Legal basis	Informed consent by patients and parents
	Environmental factors	Privacy at the patient’s home
		Adequate lighting (e.g. not too dark/no window in the background) to be seen well over webcam
		Neutral background at the therapist’s workplace
	Technical aspects	Stable high speed internet connection
		Webcam with high video resolution (at least in the range of *Full High Definition* [1080p]) Headset to avoid background noise
	Therapeutic alliance	Increased self-reflection/explanation about therapist behaviour (e.g. tell the patient when taking notes and thus not looking into the camera)
At risk patients and suicidality	Type of DHI for at risk patients	DHI without direct contact to a therapist or only asynchronous types of communication are inappropriate for patients with suicidal ideations. These comprise the risk that the therapist does not recognize suicidal ideations that would have become apparent within a personal contact
	Warning signals	Therapists have to be highly sensitive regarding key signals (e.g. severely depressed mood, hopelessness)
	Interventions in case of aggravation and safety precautions	Increase of the frequency of synchronous communication e.g. via video conferencing as a tool to deliver support during vulnerable episodes
		At least one parent has to be present in the house during video therapy sessions
		Therapist needs patient’s/parents’ contact information at hand to be able to initiate emergency services when needed
		Cooperation with local professionals (paediatrician, psychiatrists) for emergency support

**Table 2:** Benefits and pitfalls of DHI (as compared to face-to-face therapy)

Aspect	Benefits	Pitfalls
Accessibility	To (specialized) psychotherapists, especially in areas with limited availability of psychotherapists	Slow internet connection
	To psychotherapy for patients and family members with restricted mobility (e.g. somatic diseases) or fear of stigmatisation	Risk of reinforcing avoidance behaviour, e.g. for patients with the fear of leaving their house
	In times of lockdown or contact restrictions (e.g. SARS-CoV2-19 pandemic)	Barrier for patients with diminished competences in using technology, e.g. small children, older parents/caregivers, low IQ
Interventions	Broad range of methods: e.g. video conferencing, chat and e-mails, online psychoeducation, app-based interventions	Due to e.g. a webcam’s limited angular field, non-verbal behaviour (including also avoidance behaviour in therapeutic expositions) is more difficult to interpret
	Sessions with family members irrespective of their place of residence	Limited options to de-escalate emotionally difficult situations
	Facilitated possibility to conduct expositions with reaction management at the place where the problems occur	Need for involvement of a regionally located professional as backup for de-escalation
Evidence	Online psychotherapy can be as effective as face-to-face therapy	Limited evidence for some disorders and some devices such as apps
		Research gaps with regard to the influence of specific characteristics of DHI, e.g. the influence of the extent of therapeutic support within a particular DHI
		Overview of evidence-based interventions for practitioners is lacking
Economic Aspects	DHI can improve cost and time effectiveness (especially self-guided interventions or asynchronous messaging)	Initial costs for the psychotherapist (e.g. technical infrastructure)
		Reimbursement of DHI by public health insurance providers is not guaranteed in every country

In summary, DHI are promising therapeutic interventions for children and adolescents with psychiatric disorders, with already robust data regarding depression and anxiety disorders. SARS-CoV2-19 catalyses the integration of DHI into the standard repertoire of child and adolescent psychiatry and psychotherapy. Nevertheless, for using DHI in daily therapeutic work there are still challenges to overcome, ranging from technical aspects, e.g. general availability of the technical prerequisites, to the most demanding therapeutic tasks like the stabilisation of patients in acute crises. Blended treatment formats using a combination of traditional face-to-face sessions with e.g. intermittent video conferences might be the silver bullet in integrating DHI and taking advantage of their strengths.

Fortunately, at least one partner in therapy, the children and adolescents, are often accustomed to and sometimes highly trained in digitally delivered communication. So, it is up to us to use our profound expertise in traditional psychotherapy to create the digital pathways for reaching out to them. Times are changing. Let’s go.
